# Redefining surgical ergonomics: a systematic review of ergonomic outcomes in robotic urological surgery

**DOI:** 10.1007/s11701-025-03057-y

**Published:** 2025-12-22

**Authors:** Benjamin Cook, Nikhil Vasdev, Angus Luk, Prokar Dasgupta

**Affiliations:** 1 Faculty of Life Science and Medicine, King’s College London, Guy’s Campus, Great Maze Pond, SE1 1UL London, UK; 2 Hertfordshire and Bedfordshire Urological Cancer Center, Department of Urology, Lister Hospital, East and North Hertfordshire NHS Trust, Stevenage, UK; 3 Department of Urology, Freeman Hospital, Newcastle Upon Tyne Hospitals NHS Trust, Newcastle Upon Tyne, Newcastle Upon Tyne, UK; 4 King’s Health Partners, London, UK; 5 King’s College London Centre for Surgery, King’s College London, London, UK; 6 Hertfordshire Medical School, University of Hertfordshire, Hatfield, UK

**Keywords:** Robot-assisted surgery, Minimally invasive surgery, Robotic urological surgery, Ergonomics, Human factors, Musculoskeletal pain, Work strain, Systematic review

## Abstract

Urological surgeons have been shown to suffer with significant musculoskeletal (MSK) discomfort during their careers directly attributed to their work. Robot-assisted surgery (RAS) is believed to offer superior ergonomics to traditional forms of surgery but concerns still exist around their use. This systematic review collates the currently available literature on the ergonomic outcomes of RAS in urology and offers comparison to other surgical modalities. The preferred reporting items for systematic reviews and meta-analysis (PRISMA) guidelines formed the basis of this review, and the study protocol was registered in PROSPERO (CRD420250650617). A thorough database search was conducted in MEDLINE/PubMed and EMBASE, with twenty-two articles eventually included in the review. Data analysis included a narrative synthesis, and sub-group meta-analysis where data homogeneity allowed. Overall, RAS offers a more ergonomic environment for urological procedures than laparoscopic, open, or endoscopic surgery. Questionnaire-based studies demonstrated favourable use among urological surgeons, although numerous issues persist such as neck and back pain. Postural and muscular assessments similarly showed improvements in ergonomy for RAS. However, prolonged poor ergonomic joint positions, and moderate activation of upper body muscles were noted in all forms of surgery. Task load indexes demonstrated lower physical demand among RAS. While RAS offers a superior ergonomic environment to minimally invasive and open surgical techniques in urology, numerous challenges still exist. Continued study is needed, and formal ergonomic assessments should become standard protocol for any emerging robotic systems to ensure that proper ergonomy is maintained across a diverse surgical population.

**Supplementary Information:**

The online version contains supplementary material available at 10.1007/s11701-025-03057-y.

## Introduction

Work related musculoskeletal disorders (WRMSDs) represent a significant occupational concern in healthcare, with surgeons amongst the worst affected. They are subjected to longer standing hours with extended periods of repetition of fine motor movements compared with other hospital physicians during the average working day [1, 2]. Urologists are significantly affected, with up to 90% having experienced work-related musculoskeletal (MSK) strain or discomfort during their careers, most commonly in the neck (70%) or the back (66%) [3]. This discomfort is often attributed to awkward positioning in the operating room (OR), with surgical techniques such as endoscopic and laparoscopic surgery (LS) associated with significant ergonomic challenges.

Although LS has been a cornerstone of minimally invasive urology, it is frequently associated with surgeon-reported muscle strain and physical discomfort [4]. The nature of LS, due to the fulcrum effect, often requires large, ungainly movements of the upper extremities to produce small accurate movements of the surgical instruments [5]. This, in combination with the frequent upright posture of the surgeon during these procedures, leads to an ergonomically challenging environment. Robot-assisted surgery (RAS) has numerous ergonomic advantages over LS. These include an adjustable seat and console, and features such as motion scaling which minimises the need for exaggerated hand movements, thereby reducing muscular effort [6].

Despite advances in robotic surgery, its ergonomic benefits remain incompletely understood. Prior narrative reviews on surgical ergonomics have focussed on the implementation of ergonomic practices or offered an overview on the challenges faced by urological surgeons [7, 8]. However, direct comparison between RAS and other surgical techniques has not been investigated. Previous reviews have failed to undertake a formal systematic literature search, and the recent advances in novel RAS systems have not been considered. This review aims to address these shortcomings and bridges gaps in literature by conducting a systematic literature search on ergonomics comparing RAS with traditional minimally invasive and open techniques, comparing the most recent surgical systems that previous literature fails to consider. Understanding and addressing these ergonomic challenges is essential not only for surgeon well-being, but also for reducing operative fatigue, maintaining surgical performance and ultimately enhancing patient safety.

This systematic review critically analyses ergonomic outcomes of RAS and assesses its benefits over traditional forms of open and minimally invasive surgery across multiple ergonomic domains. To ensure all factors are considered, this review will include both subjective outcomes, which assess the surgeon’s perceived workload and discomfort (e.g., NASA-TLX, questionnaires), and objective outcomes, which quantify physiological stress and musculoskeletal load (e.g., surface EMG, kinematic analysis).

## Materials and methods

### Search strategy

**Fig. 1:**
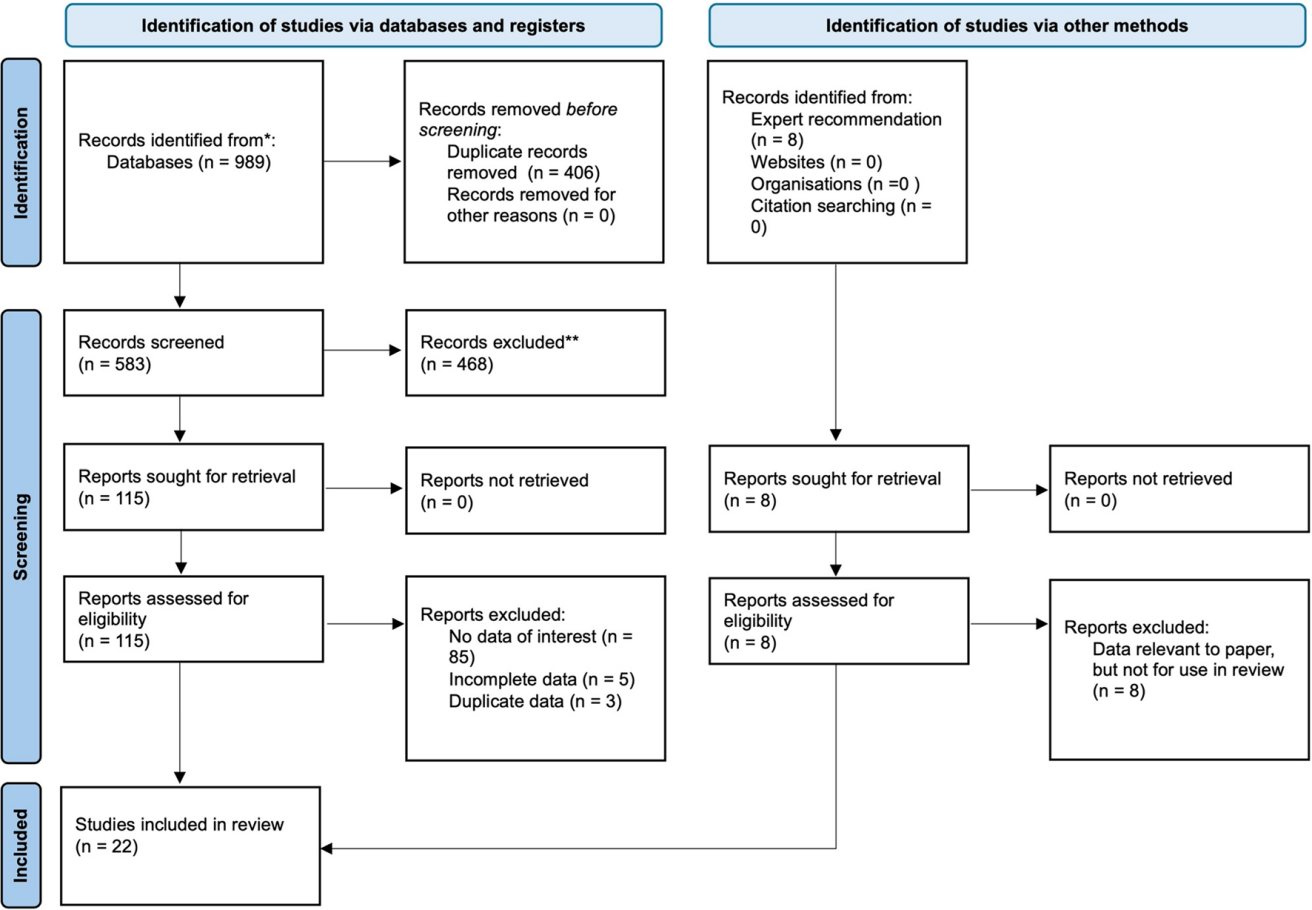
Preferred Reporting Items for Systematic Review and Meta Analyses (PRISMA) flow diagram

This review followed the Preferred Reporting Items for Systematic Reviews and Meta Analyses (PRISMA) guidelines, and its protocol was registered in PROSPERO (ID: CRD420250650617) [9]. Figure 1 outlines the strategy used. A comprehensive search of PubMed/MEDLINE and Embase was conducted in January 2025 using the following search string (“Body Position” OR “Human Factors” OR “Ergonomics”) AND (“Urol*”) AND (“Robot*”) to identify articles published over the past 25 years (Table S1). Only English-language articles were included, or where a full translated manuscript was available.

### Review of studies

Across all databases, 989 articles were returned. Duplicates were removed and all remaining manuscripts underwent abstract screening, facilitated by EndNote [10]. To minimize the risk of excluding relevant data, a low threshold for inclusion was applied during the title and abstract screening. Abstracts with any mention of ergonomics, surgeon performance, musculoskeletal strain or other human factors had their full text analysed to avoid missing data not explicitly stated in the abstract. Any ambiguity was resolved through full text screening at this stage, where all relevant data was analysed to determine its role in an ergonomic evaluation. Selected articles had their full texts analysed against the inclusion and exclusion criteria detailed in Table S2. To ensure smaller novel robotics studies were included, no minimum sample size was used. Twenty-two manuscripts were eventually included in the review. The bibliographies of included studies, along with articles recommended by the two supervising authors (N.V and P.D), were also reviewed, but no additional studies were included. The included studies were reassessed by the supervising author (N.V) for inclusion and no further changes were made.

### Data extraction

Data extraction was performed by the lead author (B.C) and included the following: (1) Author; (2) Year of study; (3) Country of study; (4) Robotic system under investigation; (5) Study design; (6) Method of ergonomic assessment; (7) Procedure under investigation; (8) Comparative surgical system; (9) Number study participants; (10) Number of performed procedures; (11) Study results. Not all studies included every datapoint mentioned, but this did not affect study inclusion unless it was felt that this compromised the analysis of the results. Studies with missing or incomplete results data were excluded from the review.

### Data analysis

Due to the substantial heterogeneity in study methodologies and statistical analyses, full meta-analysis was not feasible. Instead, a narrative synthesis was performed, grouping studies based on the ergonomic assessment used. Comparisons are made between homogenous data from separate studies. Where datasets were complete, means (M) and standard deviation (S) were calculated, if they had not been already. Random-effects model meta-analysis was undertaken where data homogeneity allowed - where S is not given, minimum values (a), maximum values (b) and sample size (n) are used to calculate S where: $\:S=\frac{b-a}{\surd\:12},\:\:if\:n\le\:15$ [11]. Meta-analysis was performed using the software Jamovi and the MAJOR plugin [12].

### Risk of bias

To assess the quality of included studies, the Risk of Bias in Non-Randomised Studies of Intervention (ROBINS-I) was used [13]. One randomised control study was included, and the RoB 2 tool was used [14]. The nature of some study designs meant ROBINS-I was not appropriate in every domain, but reasonable adjustments were made to the assessed domains to facilitate analysis. Those having moderate risk of bias in two or more domains, or one serious risk domain, were deemed of moderate risk. Those with a serious risk of bias in more than one domain were deemed overall serious risk. ROB analysis was carried out by the lead author (B.C) and checked by the supervising author (N.V). The ROBVIS tool was used to visualise the risk associated with each non-randomised study [15].

### Ergonomic assessments

Ergonomic assessments encompass a range of postural, musculoskeletal and subjective assessments on the physical strain of a task on the body of the participant and identify high-risk positioning. Assessments discussed in this review are detailed below (Table 1) [16–20].

**Table 1:** Ergonomic assessments and their description

Ergonomic Assessment	Description
Questionnaire	Participants are asked to rate their ergonomic experience across different surgical modalities. This may include items such as perceived discomfort, numbness, pain etc.
Electromyography (EMG)	EMG sensors are used to measure muscle activation during surgery. For example, the biceps brachii may have its activation measured during certain time points during the procedure.
Kinematic Sensors	Kinematic sensors measure the angles of joints in the body. For example, a kinematic sensor may be used to measure cervical flexion/extension.
Motion Analysis	Video cameras are used to measure the angles of joints in the body. For example, a camera may record the position of a surgeon’s upper limbs, and a computer program will determine the angle between the forearm and the wrist.
NASA Task Load Index (NASA-TLx)	The NASA-TLx assessment measures the perceived mental, physical and temporal demand, as well as the perceived performance, effort and frustration experienced during the task.
Surgeons’ Task Load Index (SURG-TLx)	The SURG-TLx assessment is a modification of NASA-TLx, where the final three domains are replaced with ratings of task complexity, situational stress and distraction.
Borg Scores	The Borg score is used to gauge the intensity of an activity based on the participants perceived effort and exertion.
Rapid Entire Body Assessment (REBA)	Ergonomic tool used to assess the entire body and its risk of injury.
Rapid Upper Limb Assessment (RULA)	Ergonomic tool used to assess the upper limbs and their risk of injury, often during repeated upper limb movements.

## Results

A total of 22 studies met the inclusion criteria (Table 2). Most articles included direct comparison of robotic surgery to other forms of urological surgery, most commonly standard laparoscopic surgery, but also others including open, hand-assisted laparoscopic and endoscopic surgery. A range of ergonomic assessments were utilised; Questionnaire based assessments of various forms were the most common (*n* = 13), with EMGs (*n* = 4), NASA-TLx (*n* = 4), Borg scores (*n* = 3) and numerous other postural or physical workload-based assessments making up the remainder. Heterogeneity of data prevented meta-analysis and as such, a narrative synthesis was undertaken.

**Table 2:** Study characteristics of articles included in the review

Reference	Surgical System (Manufacturer)	Study Design	Assessment Method	Procedure	*N*(*)
*Bagrodia et al.* [21]	-	Cross-Sectional	Questionnaire	Radical Prostatectomy	106
*Baumgarten et al.* [22]	-	Case-study	EMG, Kinematic Sensor, Fatigue testing	Radical Cystectomy	1
*Bigham et al.* [23]	-	Cohort Study	Kinematic Sensor, Questionnaire	-	30
*Dai et al.* [24]	KD-SR-01 (SuZhou KangDuo Robot Co., Ltd., Suzhou, China)	Randomised-Control Study	NASA-TLx, EMG	Partial Nephrectomy	1
*Elhage et al.* [25]	Da Vinci Surgical System (Intuitive Systems, Sunnyvale, CA, USA)	Repeated-Measures	Borg Score	Vesico-Urethral Anastomosis	6
*Fan et al.* [26]	KD-SR-01 (SuZhou KangDuo Robot Co., Ltd., Suzhou, China)	Prospective single-arm clinical trial	NASA-TLx	Radical Prostatectomy	1
*Fiori et al.* [27]	Ily Robot (STERLAB, Vallauris, France)	Cross-Sectional	Questionnaire	Flexible Ureteroscopy	4
*Gaboardi et al.* [28]	Da Vinci Surgical System (Intuitive Systems, Sunnyvale, CA, USA)	IDEAL Stage 1	Questionnaire	Radical Prostatectomy	1
*Giberti et al.* [29]	-	Cross-Sectional	Questionnaire	-	17 (12)
*Gofrit et al.* [30]	-	Cross-Sectional	Questionnaire	Adrenalectomy, Nephrectomy, Partial Nephrectomy, Pyeloplasty, Prostatectomy	73
*Hayashi et al.* [31].	-	Cross-Sectional	REBA scale	Radical Prostatectomy	10
*Hubert et al.* [32]	Da Vinci Surgical System (Intuitive Systems, Sunnyvale, CA, USA)	Comparative Prospective Observational	NASA-TLx, Borg score, EMG	Partial Nephrectomy	11
*Lee et al.* [33]	Da Vinci Surgical System (Intuitive Systems, Sunnyvale, CA, USA)	Cross-Sectional	Questionnaire	-	432 (88)
*Marçon et al.* [34]	Da Vinci Surgical System (Intuitive Systems, Sunnyvale, CA, USA)	Multicentre Prospective Observational	NASA-TLx, Borg score	Donor Nephrectomy	258
*Norasi et al.* [35]	Da Vinci Surgical System (Intuitive Systems, Sunnyvale, CA, USA)	Cross-Sectional	Questionnaire	-	245 (19)
*Park et al.* [36]	EasyUretero (Roen Surgical, Daejeon, South Korea)	Cross-Sectional	Questionnaire	Flexible Ureteroscopy	6
*Pérez-salazar et al.* [37]	Versius Surgical System (CMR Surgical, Cambridge, UK)	Prospective Crossover Experimental	EMG, Motion Analysis, RULA, SURG-TLx	Simulation Tasks, Nephrectomy	6
*Rassweiler et al.* [38]	Da Vinci Surgical System (Intuitive Systems, Sunnyvale, CA, USA)	Repeated-Measures	Questionnaire	Radical Prostatectomy	1
*Saglam et al.* [39]	Roboflex Avicenna (Elmed Medical System, Ankara, Turkey)	IDEAL Stage 2	Questionnaire	Flexible Ureteroscopy	7
*Saikali et al.* [40]	-	Cross-Sectional	Questionnaire	-	40 (35)
*Sánchez-Margallo et al.* [41]	Kymerax System (Terumo Europe, Leuven, Belgium)	Repeated-Measures	RULA, Motion Analysis,	Vesico-Urethral Anastomosis	7
*Tokas et al.* [42]	Da Vinci Surgical System (Intuitive Systems, Sunnyvale, CA, USA)	Repeated-Measures	Questionnaire	Radical Prostatectomy (Training tasks)	7

### Questionnaire-based assessment

Across thirteen studies, RAS demonstrated lower-self reported musculoskeletal discomfort compared to LS and OS [21, 23, 27–30, 33, 35, 36, 38–40, 42]. One study found neck pain was present in 43% of urologists, with 50% stating their neck/back pain was as a result of, or exacerbated by, operating [21]. RAS showed the lowest rate of neck and/or back pain, with 23% of urologists experiencing discomfort, compared to 50% and 56% in OS and LS respectively. Another study demonstrated RAS had the lowest rate of complaints compared to LS or hand-assisted laparoscopic surgery (HALS), with < 5% experiencing upper back pain, numbness or fatigue, < 5% experiencing shoulder pain or hand/wrist fatigue or numbness [30]. Finger pain was the most common complaint associated with RAS (> 10%). While not specific to urology (19/79 urologists), The Da Vinci system demonstrated significant improvements in perceived physical strain compared to OS and LS (Physical demand Med: RAS = 3 vs. OS = 7 and LS = 5; Physical Strain Med: RAS = 3 vs. OS = 5 and LS = 3) [35]. Endoscopic surgery showed similar physical demands to RAS. The lowest rates of neuromusculoskeletal pain were also noted in RAS (Overall: 21%; Upper Extremity: 14%; NMSDs: 7%), particularly in the shoulders and the fingers of the left hand.

The EasyUretero system showed significant ergonomic improvements over HALS in one study, with reductions in shoulder and wrist stiffness, and thumb and hand fatigue [36]. A 2 to −2 rating score was used, defined as the following: (2) very good; (1) good; (0) no difference; (−1) poor; (−2) very poor. Experts, fellows and residents all reported improvements in shoulder stiffness (0.50 ± 0.84, 0.83 ± 0.41, 0.40 ± 0.55 respectively), with experts and residents also noting improvements in thumb fatigue (1.50 ± 0.55 and 1.80 ± 0.45) and hand fatigue (0.83 ± 0.41 and 1.60 ± 0.55). Fellows did not note improvements in thumb or hand complaints (0.00 ± 0.89 and 0.00 ± 0.63 respectively) and noted worsening wrist stiffness in comparison to HALS (−0.50 ± 0.55).

Two studies investigated the ETHOS ergonomic system for LS, comparing it to traditional LS and RAS with the Da Vinci system [38, 42]. Both noted improvements in ergonomy when using the ETHOS system but fell short of the exceptional ergonomic performance of the Da Vinci. Total complaint scores, defined as the sum of the mean complaints received for each body area, each ranked on a 0–5 (no discomfort - severe pain) scale, in the first study showed the Da Vinci significantly ahead of LS (M:4.5 vs. 31.6), and modest improvements over ETHOS (M:13.9) [38]. Mean complaints per body area did not exceed 1 for the Da Vinci, with many areas experiencing no discomfort (0). The second study delivered a more detailed analysis of ETHOS, assessing its ergonomy through multiple degrees of freedom (DOF) across three tasks of increasing difficulty, again in comparison to traditional LS and the Da Vinci system [42]. The Da Vinci again proved its ergonomic superiority, with 86.7%, 100% and 73.3% of participants reporting no/low discomfort during the three simulated tasks. ETHOS demonstrated impressive results compared to LS, with increasing DOF and the use of a 3D laparoscopic camera reducing ‘heavy’ discomfort to nil in the first two tasks, and only 6.7% in the final task. However, ETHOS again failed to match the Da Vinci’s exceptional comfort.

RAS was not without complaints, with three studies identifying significant MSK distress among urological robotic surgeons as a result of the hunched neck position in the console. A study comprising a significant proportion of urological surgeons (70.5%) identified 41.2% experiencing recurrent MSK pain since starting robotic surgery, with 35.3% experiencing it on a daily basis [29]. Pain in the cervical spine and upper limbs were most common (29.4% and 23.5% respectively). In another study, 70% of urology respondents reported discomfort in RAS [33]. Eyestrain was particularly common, with increasing years of practice correlating with worsening eyestrain, although eye fatigue was noted to be significantly decreased in subsequent generations of the Da Vinci system (standard, S and Si). Regarding body areas, 22.5% of respondents reported finger fatigue, and 21.4% neck stiffness. Upper body discomfort continued in the final study, with 70% reporting pain in their shoulders, 35% pain in their neck, and 25% pain in their face and head [40]. Over half (52.5%) directly correlated the perceived discomfort with robotic surgery, and as with the previous study, case length and number were theorised to play a bigger role in physical strain than the ergonomy of the robotic console.

### EMGs and postural assessments

Nine studies investigated postural and muscular parameters during robotic procedures. Those utilising EMGs (*n* = 4) demonstrated significantly lower overall muscle activation during RAS compared to other surgical techniques [22, 24, 32, 37]. Heterogeneity prevented statistical analysis, so the following table outlines muscle groups and their activation in RAS and LS or OS across three studies (Table 3). For ease of interpretation, estimated % Maximum Voluntary Contraction (MVC) will be coded using the following syntax: <20%MVC – Low; 20–40%MVC – Medium; >40%MVC - High. Mean %MVC will be estimated where multiple data points are used.

**Table 3:** EMG analysis of upper and lower body muscles during urological surgery^1^

**Muscle**	**Pérez-Salazar** *et al.*		**Hubert** *et al.*		**Baumgarten** *et al.*	
	RAS	LS	RAS	LS	RAS	OS
***Upper Body***						
**R-TRAP-UP**	Low	Low	Low	Medium	Medium	Medium
**L-TRAP-UP**	Low	Low	Low	Medium	Medium	Low
**R-TRAP-MID**	Low	Medium			Medium	Medium
**L-TRAP-MID**	Low	Low			Medium	Low
**R-BRACH**	Low	Low				
**L-BRACH**	Low	Low				
**R-ER-SPIN**	Low	Low	Low	Medium		
**L-ER-SPIN**	Low	Low	Low	Medium		
**R-TRI-BRACH**	Low	Low				
**L-TRI-BRACH**	Low	Low				
**R-VAS-LAT**	Low	Low				
**L-VAS-LAT**	Low					
**R-DELT**					Low	Low
**L-DELT**					Low	Low
**R-INFRA**					Low	Medium
**L-INFRA**					Medium	High
***Lower Body***						
**R-PARA**					Low	Medium
**L-PARA**					Low	Medium
**R-REC-FEM**					Low	Low
**L-REC-FEM**					Low	Low
**R-GAS**	Low	Low			High	Medium
**L-GAS**	Low	Low			Low	Medium
**R-TIB-ANT**					Low	Low
**L-TIB-ANT**					High	Medium

^1^**Abbreviations:** Right upper trapezius (R-TRAP-UP); Left upper trapezius (L-TRAP-UP); Right middle trapezius (R-TRAP-MID); Left middle trapezius (L-TRAP-MID); Right Brachialis (R-BRACH); Left brachialis (L-BRACH); Right erector spinae (R-ER-SPIN); Left erector spinae (L-ER-SPIN); Right vastus medialis (R-VAS-MED); Left vastus medialis (L-VAS-MED); Right triceps brachialis (R-TRI-BRACH); Left triceps brachialis (L-TRI-BRACH); Right vastus lateralis (R-VAS-LAT); Left vastus lateralis (L-VAS-LAT); Right deltoid (R-DELT); Left deltoid (L-DELT); Right infraspinatus (R-INFRA); Left infraspinatus (L-INFRA); Right paraspinal muscles (R-PARA); Left paraspinal muscles (L-PARA); Right rectus femoris (R-REC-FEM); Left rectus femoris (L-REC-FEM); Right gastrocnemius (R-GAS); Left gastrocnemius (L-GAS); Right tibialis anterior (R-TIB-ANT); Left tibialis anterior (L-TIB-ANT); Robot-assisted surgery (RAS); Laparoscopic surgery (LS); Open surgery (OS)

Borg scores (*n* = 3) demonstrated favourable results across the board for RAS, particularly in comparison to LS [25, 32, 34]. The first study by Elhage et al. showed significantly lower Borg scores in RAS (Med: 1) than LS (Med: 3) [25]. While one-way ANOVA demonstrated improvements over LS to be significant (*P < 0.005*), improvements over OS were not statistically significant (*P = 0.272*). Body area analysis was not provided. These findings were echoed in a second study, with RAS scoring significantly lower across all body areas than LS (*P < 0.005*), most notably the shoulders (RAS: 1.6 ± 0.2 vs. LS: 3.1 ± 0.2), neck (RAS: 1.7 ± 0.2 vs. LS: 3.1 ± 0.2) and the back (RAS: 1.8 ± 0.2 vs. LS: 3.1 ± 0.2) [32]. RAS’s superior ergonomics were again highlighted in a final study assessing Borg scores for both the lead surgeon, and surgical assistant [34]. Multiple body areas demonstrated extremely low exertion at varying time points (Left shoulder/arm M: 0.07 ± 0.34; Left forearm/hand M: 0.00 ± 0.15; Right shoulder/arm M: 0.10 ± 0.42; Lower back M: 0.80 ± 0.92). OS, HALS and LS all fell short of the superior ergonomy of RAS. The ergonomics of the surgical assistant were less favourable for RAS compared to other surgical modalities - RAS demonstrated higher exertion in multiple body areas at varying time points (Neck M: 0.60 ± 1.16; Right shoulder/arm M: 0.68 ± 1.08; Right forearm/hand M: 0.54 ± 1.05; Lower back M: 0.98 ± 1.18). However, overall exertion was still low.

Four studies investigated posture through analysis of join positioning (i.e. flexion/extension, abduction/adduction etc.), through either kinematic sensors (*n* = 2) or motion capture analysis (*n* = 2) [22, 23, 37, 41]. The first identified that during RAS, 100% of the time during radical cystectomy was spent in cervical flexion [22]. Of note, OS did not demonstrate major improvements, with 95%. Neck flexion was also analysed in another study, showing contradicting results to previous [23]. RAS was noted to have lower time spent in neck flexion (> 30º) compared to OS (68 min vs. 147 min), although neck extension was noted to be similar (54 min vs. 57 min). Motion capture allowed for comparison of multiple body areas between conventional LS and RAS, but consensus was unclear on what modality offered better ergonomic performance. The first demonstrated LS offered better ergonomy than RAS in numerous body areas including neck flexion/extension, back flexion/extension and shoulder flexion/extension [37]. Shoulder abduction/adduction showed improvements in RAS, while others demonstrated no significant difference between the two modalities. The lack of published data limited analysis. The final study utilised both motion capture and a ‘data glove’ (Cyber Glove), to assess various hand postures [41]. RAS showed improvements in elbow extension (RAS: 120.63 ± 13.13º vs. LS: 134.68 ± 14.35º) and shoulder flexion (RAS: 18.48 ± 14.17º vs. LS: 26.12 ± 7.41º), although no statistically significant differences were noted elsewhere. ‘Data glove’ assessment showed varying results, with no modality performing better than the other.

Studies using the REBA/RULA ergonomic risk tools (*n* = 3) demonstrated significantly lower scores in RAS compared to other surgical modalities [31, 37, 41]. RAS scored significantly lower in the REBA assessment during one study, with intraindividual (*P < 0.0001*) and grouped analysis (RAS: 3.02 ± 0.32 vs. LS: 4.63 ± 0.21) both showing marked improvements over LS [31]. REBA action level did not exceed medium risk in RAS (100% low/medium risk). However, the Versisus surgical system did not show any improvements in RULA score compared to LS, in either upper limbs (RAS: 3 vs. LS: 3) or body/lower limbs (RAS: 3 vs. LS: 3) during total nephrectomy [37]. Global score similarly did not show any improvements (RAS: 4 vs. LS: 4). Finally, a modified RULA (mRULA) assessment was used to analyse wrist posture between LS and RAS (Kymerax system) [41]. Both surgical modalities demonstrated acceptable (mRULA score 1–2) wrist posture during the first ureterovesical anastomoses, but this progressed to unacceptable (mRULA score 3) by the final repetitions. No modality outperformed the other.

### NASA-TLx and workload assessments

**Fig. 2:**
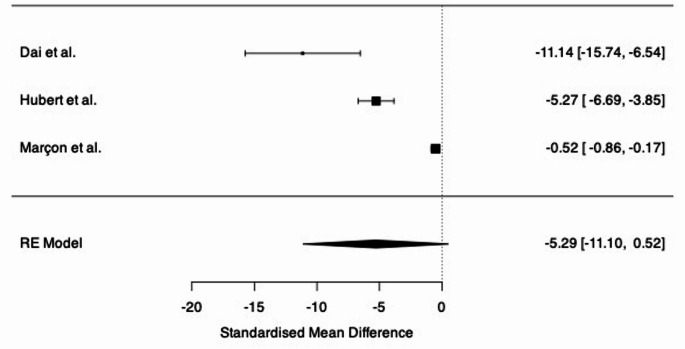
NASA-TLx random-effects model meta-analysis - Forest plot summarising effects estimate and confidence intervals for RAS versus LP across three studies. The central squares represent the effect size for each study, and the horizontal bars represent the 95% confidence intervals. The diamond represents the overall pooled estimate

Five studies investigated general workload during robotic procedures, including physical and mental workloads, through the use of the NASA-TLx (*n* = 4) or SURG-TLx (*n* = 1) assessment criteria [24, 26, 32, 34, 37]. The KD-SR-01 robotic system demonstrated significant ergonomic improvements over 3D LS, both based on NASA-TLx physical demand (PD) and global score (GS) (RAS PD: 3.00 vs. LS PD: 5.75, *p* < 0.05; RAS GS: 22.75 vs. LS GS: 34.50, *p* < 0.05) [24]. All domains scored lower for RAS than LS. A later study assessing the same surgical system also demonstrated favourable NASA-TLx scores during RARP, both in PD (5.5 ± 0.8) and GS (22.7 ± 3.2), although no comparison was offered [26]. The Da Vinci system also proved ergonomically beneficial for LS, with lower PD scores than standard LS (RAS: 3.0 ± 0.5 vs. LS: 5.7 ± 0.5) [32]. However, a second study showed that while physical demand continued to be lower in the Da Vinci compared to LS, HALS and OS (RAS: 41.8 ± 16.4 vs. LS: 52.3 ± 23.6 vs. HALS: 53.2 ± 28.2 vs. OS: 54.6 ± 25.4), overall GSs were highest in RAS (RAS: 73.9 ± 15.0 vs. LS: 65.3 ± 20.1 vs. HALS: 48.3 ± 24.4 vs. OS: 72.1 ± 20.6) [34]. Random-effects model meta-analysis across three studies demonstrated lower NASA-TLx scores in RAS than LS, although did not reach statistical significance (SMD: −5.29 [−11.1 to 0.52], *p* = 0.074) (Fig. 2) [24, 32, 34]. A surgery-specific workload assessment (SURG-TLx) was utilised by one study and demonstrated lower physical demand in RAS than LS [37].

### Risk of bias

A low ROB was found in eight studies. Fourteen demonstrated moderate ROB, with low risk in most domains. No study was found to be of critical risk. Four studies were deemed high risk of participant selection bias due to involving only a single surgeon. ROBINS-I analysis is detailed in Table S4 and Figure S1. One randomised-control study demonstrated moderate risk of bias due to the lack of blinding and the use of a subjective assessment.

## Discussion

This systematic review identified 22 studies investigating ergonomic outcomes of robot-assisted urological surgery, with evidence demonstrating reduced musculoskeletal pain, improved posture and lower subjective workloads compared to other surgical modalities such as laparoscopic and open surgery. Despite strong evidence in favour of robotic surgery, many ergonomic challenges persist, particularly around neck, shoulder and upper back strain from prolonged console use. Newer surgical systems such as the EasyUretero and Versius system have demonstrated ergonomic profiles similar to the gold-standard seen in Da Vinci, and upcoming systems will continue to iterate to provide a superior human-centred work environment.

Questionnaire-based assessment highlighted that most surgeons prefer the ergonomy seen in robotic surgery. However, there were still large numbers of surgeons experiencing discomfort in the neck, back and shoulders, and while often lower than LS and OS, this still poses a significant challenge for urological surgeons [21, 23, 29, 30, 33, 35, 40, 43]. Studies utilising other subjective assessments like Borg scores and REBA/RULA assessments showed similar findings [25, 31, 32, 34, 37, 41]. However, this was dependent on the surgical system being investigated. The Versius system showed no significant differences to standard LS under the RULA assessment, nor did the Kymerax system, which also highlighted that ergonomic stressors could change dynamically throughout the surgery [37, 41]. Assessments taken at the end of the surgery demonstrated significantly more strain than earlier in the procedure, and future studies should ensure that multiple time points are used when assessing ergonomic outcomes. While subjective assessments can provide a strong insight into the perceived discomfort associated with surgery, their data should always be analysed with a level of scrutiny owing to their moderate risk of self-reporting bias. Nonetheless, the perceptions of surgeons in their working environment should still be considered alongside more objective ergonomic assessments.

Objective assessments such as EMGs and posture analysis similarly demonstrate the ergonomic advantages of robotic surgery but also highlight additional challenges. Muscle activation in RAS was deemed low across most muscle groups in most studies and often offered lower activation than other surgical modalities [22, 32, 37]. The trapezius and the lower back muscles demonstrated the largest differences, likely due to the seated versus standing posture seen in RAS compared to LS or OS. Lower body muscles also showed lower overall activation in RAS than OS, although RAS did lead to abnormally high activation in the right gastrocnemius and the left tibialis anterior [22]. While the reason for this is unclear, the use of foot pedals in RAS could play some role. Notably, analysis of join positions demonstrated the concerning neck extension/flexion in RAS, likely due to the use of the binocular lenses at the surgical console [22, 23]. Repeated anterior/posterior movement of the neck when switching between looking at the console and communicating with the wider surgical team also likely resulted in increased neck strain during procedures.

Considering workload assessments, RAS repeatedly offered lower physical demand than LS, OS or HALS. Meta-analysis of three NASA-TLx studies confirmed RAS’ physical ergonomics to be vastly superior to LS, and while the random-effects model analysis did not reach statistical significance (*p* = 0.074), individual study analysis did [24, 32, 34]. The nature of task-load assessments did not allow for per-body-part analysis but instead offered insight into the ergonomics overall. Notably, NASA-TLx highlighted a challenge not often investigated by ergonomic studies – cognitive ergonomics. Mental and temporal demand (i.e. the perceived stress on the mind and time pressure) were significantly higher in RAS, suggesting that while the physical environment may be ergonomically superior, this may be at the cost of stress in other areas [16]. However, previous studies have not shown a higher cognitive demand in robotics, most in fact showing the opposite, so this result should be considered carefully [44].

Assessing both subjective and objective measures of ergonomics together demonstrated both convergent and divergent findings, suggesting that there are cases where individual assessment tools should not be considered conclusive alone. Findings converged strongly regarding upper-extremity ergonomics. Both subjective reports and objective data consistently demonstrated that RAS reduces physiological load on the distal upper limbs compared to laparoscopic (LS) and open surgery (OS). Subjective improvements in hand and wrist fatigue were corroborated by objective reductions in EMG muscle activation and lower Borg ratings for shoulder and arm exertion [22, 24, 25, 30, 32, 34, 36, 37, 41]. This alignment between perception and physiological metrics suggest that the ergonomic design of robotic consoles effectively offloads the surgeon’s upper extremities. However, a significant divergence was observed regarding cervical ergonomics. While subjective surveys generally favoured RAS with lower reported rates of neck pain compared to OS and LS, objective kinematic data revealed a persistent ergonomic hazard [21, 22, 32, 37]. Motion analysis indicated that RAS surgeons may spend up to 100% of the operative time in cervical flexion, with some motion capture data suggesting that LS may actually offer superior neck posturing [22]. This suggests that while the perception of neck strain is reduced in the robotic console, potentially due to increased comfort in other body areas, the physiological static load on the cervical spine remains high.

### Remaining challenges in robotic surgery

Although RAS offers an environment more ergonomic than other forms of minimally invasive or open surgery, there are still numerous challenges that need to be addressed. A 2017 article investigating the ergonomics of the Da Vinci showed risk-prone neck positioning, using similar assessment methods described in this review [45]. Concerning neck positions were noted in all participants, and this aligns with the literature discussed previously. This paper also highlighted unergonomic positioning of the shoulders and wrists, although the inclusion of a wrist rest likely mitigates some of this risk [46, 47]. The cause of this is unclear although it has been theorised that the lack of haptic feedback may lead to surgeons applying excessive force to the robot manipulators, leading to the increased wrist and finger strain seen in these studies [48]. Eye strain has also been noted to be one of the downsides of RAS, mostly due to the 3D visuals used, compared to the 2D visuals used in LS [49]. Quantifying this is challenging, and it is not known if there are any permanent effects on surgeons’ vision, although questionnaires have noted short-term eye strain, or ‘computer vision syndrome’ [50]. Some of these limitations may be better addressed by open consoles such as the Versius and Hugo systems and the DaVinci5 where the surgeon is also seated upright, although continued study is needed.

Sex, size and anthropometric differences between surgeons also plays a large role in surgical ergonomics. For example, women have been shown to have significantly higher muscle activation than men during simulated ureteroscopy, as well as higher global and physical demand scored in NASA-TLx [51]. Endoscopic procedures have also been associated with greater back pain in women than in men [52]. Regarding robotics, it has been theorised that women may experience higher rates of strain and a poorer overall experience in RAS, perhaps due to reduced muscle strength [53, 54]. In other forms of minimally invasive surgery, smaller hand sizes, shorter stature and lower muscle mass also likely play a role in the differences seen [55]. The adjustability of robotic consoles likely mitigates some of these challenges, but prolonged unergonomic positioning may still lead to high rates of muscle strain and discomfort. Console adjustability is also only effective within certain constraints, with surgeons between 64 and 73 inches (163–185 cm) tall the only group able to adhere to ergonomic recommendations of the Da Vinci (note average female height of 162.4 cm in England; outside the limits of the Da Vinci) [56, 57]. This can be mitigated by placing the foot pedals on an elevated surface, although this is only possible in consoles with detachable foot pedals [6]. Beyond anthropometrics, age and experience also affect ergonomics, although these differences are harder to overcome.

The are multiple proposals of ways to alleviate physical strain during surgery, although most typically focus on non-robotic forms of minimally invasive surgery. For robotics, techniques typically address proper ergonomic posture or use add-on devices that offer improvements in body position or ergonomic adherence. An alarm was added to the Da Vinci system that would signal inappropriate use of the armrest and demonstrated faster acquisition of an ergonomic position during simulated tasks [58]. Proper set-up of the surgical console is instrumental in ensuring that proper ergonomy is maintained. This includes ensuring that the viewing angle is approximately 15°, ensuring light pressure on the headrest, avoiding neck flexion beyond 25°, avoiding back flexion beyond 15°, adjusting the seat to popliteal height, keeping knees at right angles and prevent excessive foot dorsiflexion when using pedals, and elbows should be kept by the surgeons’ sides and remain at right angles [59]. Tetteh et al. have suggested a formal step-by-step set-up guide for the Da Vinci system to adhere to these recommendations [60]. Eye strain can be effectively mitigated by correcting refractive errors, taking short screen breaks, and ensuring eyes don’t become dry with eye drops and blinking efficiency training [61]. Micro-breaks, ergonomic training and resistance training are recommended to reduce musculoskeletal load during RAS, although some recommendations such as more robotic experience cannot be implemented in the short term. Micro-breaks (short, regular pauses during procedures for stretching) in particular have been shown to substantially reduce surgeon pain and improve overall performance, including significantly reducing intraoperative errors [62]. Reminders for micro-breaks may be required, using apps such as OR-Stretch™, to ensure that stretch breaks are adhered to [63]. These recommendations should be implemented where possible into routine surgical practice, and their effects appropriately investigated.

### Limitations

This review did have some limitations. The primary limitation was the lack of meta-analysis due to methodological heterogeneity. Only sub-group meta-analysis was undertaken and reported data had to be manipulated to allow for comparative analysis. Despite the use of other semi-standardised methodologies such as Borg scores and REBA/RULA assessments, data heterogeneity meant that full meta-analysis could not be undertaken. The same was true of EMG and joint analysis. Many studies did not present raw data, instead opting to report only on key statistics which may have introduced significant reporting bias. In studies with small sample sizes, many investigated only one surgeon, and while different patients were used, the same procedure was often undertaken, limiting applicability of the data to the wider urological community. Small sample sizes are an inherent limitation of ergonomics research and limit the generalisability of each study individually, making blanket recommendations challenging when accounting for the significant methodological differences seen in studies with larger sample sizes. Additionally, as reported in the risk of bias, this introduced significant participant selection bias, although it was not felt to affect their inclusion in the review. The final limitation of this study was the lack of articles investigating new robotic systems. A significant proportion of those that detailed the robotic system in use, used the Da Vinci system. While this is the most widely used system worldwide, ergonomic assessments of novel robotic systems coming to market would have offered a prospective insight into the future of ergonomics in RAS and may have identified further improvements required for the next generation of robotic systems.

Despite some limitations, this review concludes that RAS can offer a superior ergonomic environment to other forms of surgery, although numerous challenges persist. Implementation of the discussed recommendations can help alleviate some ergonomic problems associated with RAS, although continued investigation is needed to better quantify the effects these interventions have. Standardised objective assessment measures and consideration for more diverse surgeon populations will be crucial in advancing surgical ergonomics and optimising robotic systems.

## Conclusion

Robotic surgery in urology offers surgeons a more ergonomic and comfortable operating environment across a range of procedures and surgical systems, although numerous challenges persist. Muscle and joint stress are still common in areas such as the neck and upper back, with the repetitive movements seen in RAS a potential risk factor for repetitive strain injuries and prolonged musculoskeletal discomfort. Subjective analysis demonstrated an overall positive outlook from robotic surgeons, but again many still note strain and discomfort when operating. Future iteration in surgical robotics should aim to mitigate the remaining stressors, and formal ergonomic assessment should become a mainstream consideration for all novel robotic systems coming to market. Ergonomic training should be formally integrated into the surgical curriculum to ensure adherence to proper ergonomic practices, and techniques utilised to reduce both the physical and mental stress associated with robotic surgery in urology. WRMSDs will likely persist in urology for the foreseeable future, but any means of mitigating this risk should be promptly incorporated into routine surgical practice and prevent further damage to the current or future surgical workforces.

## Supplementary Information

Below is the link to the electronic supplementary material.

Supplementary Material 1

## Data Availability

All data included in this review was sourced from previously published works. No additional data was collected. All new data calculated is present in the review. The rights to the data quoted in this review remain with the authors/publisher of the included articles.
