# Supplementary material for: Redefining surgical ergonomics: a systematic review of ergonomic outcomes in robotic urological surgery
Source: J Robot Surg. 2025 Dec 22;20(1):107. doi: 10.1007/s11701-025-03057-y (PMC12722418; doi:10.1007/s11701-025-03057-y)
Supplement: Supplementary file 1 — Supplementary Material 1 [file 11701_2025_3057_MOESM1_ESM.docx]

| **Database** | **Search Terms** | **Filters** | **N** |
| --- | --- | --- | --- |
| **MEDLINE/PubMed** | 1. “Body Position” OR “Human Factors” OR “Ergonomics” 2. “Urol*” 3. “Robot*” 4. 1 AND 2 AND 3 | Past 25 Years  English | 395 |
| **EMBASE (Ovid)** | 1. “Body Position” OR “Human Factors Research” OR   “Ergonomics”   1. “Urology” OR “Urological Surgery” OR “Urologist” 2. “Robot” OR “Robot assisted surgery” 3. 1 AND 2 AND 3 | Past 25 Years  English | 594 |

**Supplementary Table 1.** Full database search strategy

| Inclusion Criteria | Exclusion Criteria |
| --- | --- |
| Primary sources only | Secondary sources (e.g. reviews) |
| Studies in English or where an English translation is available | Studies with data deemed not specific to urology |
| Studies with a direct relevance to the field of urology | Studies where ergonomics is not appropriately investigated |
| Studies involving robotic surgery or robot-assisted laparoscopic surgery | Studies with incomplete or missing data |
| Studies discussing or undertaking a formal assessment of ergonomics |  |

**Supplementary Table 2.** Inclusion and exclusion criteria for included studies.

| Reference | Surgical System (Manufacturer) | Study Design | Assessment Method | Procedure | Comparison (Y/N) | N(*) | Conclusion |
| --- | --- | --- | --- | --- | --- | --- | --- |
| *Bagrodia et al.[21]* |  | Cross-Sectional | Questionnaire | Radical Prostatectomy | Y | 106 | Overall, 23% of surgeons reported neck and/or back pain in robotic surgery compared with 50% and 56% in open and laparoscopic surgery respectively. Over half (57%) ranked robotic as the most comfortable. |
| *Baumgarten et al.[22]* |  | Case-study | EMG, Kinematic Sensor, Fatigue testing | Radical Cystectomy | Y | 1 | EMG analysis demonstrated decreased average muscle activation across most major muscle groups in RAS compared to OS. Lower leg muscle EMG demonstrated increased activation in some groups during RAS, and 100% of the surgery was spent in cervical flexion. Mild improvements were noted in fatigue testing between RAS and OS. |
| *Bigham et al.[23]* |  | Cohort Study | Kinematic Sensor, Questionnaire |  | Y | 30 | Overall, 147 minutes was spent in cervical flexion exceeding 30° during OS, versus 68 minutes during RAS. Neck pain was noted to be lower after RAS. |
| *Dai et al. [24]* | KD-SR-01 (SuZhou KangDuo Robot Co., Ltd., Suzhou, China) | Randomised-Control Study | NASA-TLx, EMG | Partial Nephrectomy | Y | 1 | SMW of the right FCU and left BB was significantly higher in LS than in RAS. Significantly higher NASA-TLx scores across all six domains in LS than RAS (p < 0.05). |
| *Elhage et al.[25]* | Da Vinci Surgical System (Intuitive Systems, Sunnyvale, CA, USA) | Repeated-Measures | Borg Score | Vesico-Urethral Anastomosis | Y | 6 | One way ANOVA demonstrated significant decreases in Borg score in RAS compared to LS (median: 1 vs 3, p<0.005). OS Borg scores were higher than RAS, but not statistically significant. |
| *Fan et al.[26]* | KD-SR-01 (SuZhou KangDuo Robot Co., Ltd., Suzhou, China) | Prospective single-arm clinical trial | NASA-TLx | Radical Prostatectomy | N | 1 | Physical demand of the KD-SR-01 system was 5.5±0.8 in NASA-TLX from 1 surgeon across 16 procedures. Across all domains the system scored 22.7±3.2 (medium workload). |
| *Fiori et al.[27]* | Ily Robot (STERLAB, Vallauris, France) | Cross-Sectional | Questionnaire | Flexible Ureteroscopy | N | 4 | Median ergonomics score of 5/5 in the questionnaire across all participants. The robot was also noted to be easy to use (4/5). |
| *Gaboardi et al.[28]* | Da Vinci Surgical System (Intuitive Systems, Sunnyvale, CA, USA) | IDEAL Stage 1 | Questionnaire | Radical Prostatectomy | N | 1 | Narrative assessment of ergonomy in R-LESS-RP from both the surgeon and operating assistant demonstrated favourable ergonomics, even compared to standard RARP. |
| *Giberti et al.[29]* |  | Cross-Sectional | Questionnaire |  | N | 17 (12) | Of those responding to the survey, a large proportion were urological surgeons (70.5%). A significant proportion of surgeons experienced recurrent MSK pain since their first robotic surgery (41.2%), and 35.3% experienced MSK pain during their daily surgical activity. The cervical spine (29.4%) and upper limbs (23.5%) were most commonly affected, although the surgical console was noted to be comfortable for many (64.7%). |
| *Gofrit et al.[30]* |  | Cross-Sectional | Questionnaire | Adrenalectomy, Nephrectomy, Partial Nephrectomy, Pyeloplasty, Prostatectomy | Y | 73 | RALS was compared to LS and HALS. Across all surgeons, 30% experienced neuromuscular or arthritic injuries during surgery. RALS was associated with the fewest complains, and HALS the most. Neck complains were most common after SLS. A high rate of hand and wrist pain HALS in 37% of surgeons, and 45% experienced numbness. Back and elbow discomfort were also noted during HALS. |
| *Hayashi et al.[31]* |  | Cross-Sectional | REBA scale | Radical Prostatectomy | Y | 10 | Grouped analysis demonstrated significantly lower REBA posture scores in RAS (3.02±0.32) compared to LS (4.63±0.21). Intraindividual analysis also demonstrated significantly lower REBA scores in RAS (p< 0.0001 for all participants). REBA action level demonstrated mostly low-risk for RAS, and medium-low-risk for LS. |
| *Hubert et al.[32]* | Da Vinci Surgical System (Intuitive Systems, Sunnyvale, CA, USA) | Comparative Prospective Observational | NASA-TLx,  Borg score, EMG | Partial Nephrectomy | Y | 11 | NASA-TLX scores for physical demand was significantly lower in RAS than LS (3.0±0.5 vs 5.7±0.5 respectively), but no other domain showed any differences. Borg scores demonstrated significantly lower physical strain in the RAS procedures across all body areas. The EMG studies showed lower %MVC in all body areas studies in RAS. |
| *Lee et al.[33]* | Da Vinci Surgical System (Intuitive Systems, Sunnyvale, CA, USA) | Cross-Sectional | Questionnaire |  | N | 432 (88) | Survey reported the highest rate of strain during robotic surgery (70%). While not specific to urology, RAS did have some drawbacks, notably eye strain from the 3D image, unlike the 2D image seen in LS. Ergonomic features were noted to be helpful (M: 3.97/5). Over half (56.1%) of respondents experienced physical symptoms or discomfort. Finger fatigue and neck stiffness were noted in 22.5% and 21.4% of respondents respectively. |
| *Marçon et al.[34]* | Da Vinci Surgical System (Intuitive Systems, Sunnyvale, CA, USA) | Multicentre Prospective Observational | NASA-TLx, Borg score | Donor Nephrectomy | Y | 258 | Borg scores demonstrated significantly lower strain in the right and left lower limbs during RAS, compared to LS, HALS, and OS. However lower back exertion was highest in RAS. NASA-TLx showed the lowest physical demand in RAS (41.8±16.4 vs HALS: 53.2±28.2; LS: 52.3±23.6; OS: 54.6±25.4), but the lowest global workload score in HALS. RAS also demonstrated greater strain in the surgical assistant compared to other approaches. |
| *Norasi et al.[35]* | Da Vinci Surgical System (Intuitive Systems, Sunnyvale, CA, USA) | Cross-Sectional | Questionnaire |  | Y | 245 (19) | Physical demand and physical discomfort were lowest in RAS. Robotic surgeons were also least likely to have neuromusculoskeletal pain, physical discomfort or pain in upper extremity, or any neuromusculoskeletal disorder (21%, 14% and 7% respectively). RAS demonstrated the lowest upper extremity discomfort and the highest percentage of surgeons reporting no discomfort. |
| *Park et al.[36]* | EasyUretero (Roen Surgical, Daejeon, South Korea) | Cross-Sectional | Questionnaire | Flexible Ureteroscopy | Y | 6 | Pain and fatigue in shoulders, wrists, thumbs and hands were almost all reduced in RAS compared to HALS. The only exception was a perceived increase in discomfort of wrist in surgical fellows, and no difference was observed in thumb or hand discomfort. |
| *Pérez-salazar et al.[37]* | Versius Surgical System (CMR Surgical, Cambridge, UK) | Prospective Crossover Experimental | EMG, Motion Analysis, RULA, SURG-TLx | Simulation Tasks, Nephrectomy | Y | 6 | SURG-TLx demonstrated significantly lower physical demand in RAS than LS. Localised muscle fatigue was also lower in RAS, and during nephrectomy RAS demonstrated smaller movements in wrist flexion/extension, right shoulder abduction/adduction and left shoulder flexion/extension. LS demonstrated less movement in other body areas. |
| *Rassweiler et al.[38]* | Da Vinci Surgical System (Intuitive Systems, Sunnyvale, CA, USA) | Repeated-Measures | Questionnaire | Radical Prostatectomy | Y | 1 | The Da Vinci System consistently ranked as the most comfortable, even compared to the novel ETHOS LS ergonomic tool. RAS had the lowest overall discomfort score (4.5) compared to ETHOS (13.9) and LS (31.6) and often scored zero (no complaints) for numerous body areas. |
| *Saglam et al.[39]* | Roboflex Avicenna (Elmed Medical System, Ankara, Turkey) | IDEAL Stage 2 | Questionnaire | Flexible Ureteroscopy | Y | 7 | Far fewer complains of pain, stiffness or strain across numerous body areas were reported in RAS compared to LS. Average overall score across all seven surgeons was significantly lower than in RAS than LS (5.6±2.4 vs 31.3±7.8). |
| *Saikali et al.[40]* |  | Cross-Sectional | Questionnaire |  | N | 40 (35) | 70% reported pain in their shoulders, 35% pain in their neck, and 25% in their face and head. Most discomfort rated 5/10 or less. 52.5% believed robotic surgery to be the cause of their discomfort. Number and length of cases was believed to have more of an impact on the discomfort felt rather than the ergonomics of the robot. |
| *Sánchez-Margallo et al.[41]* | Kymerax System (Terumo Europe, Leuven, Belgium) | Repeated-Measures | RULA, Motion Analysis, | Vesico-Urethral Anastomosis | Y | 7 | During the beginning stages of the procedure, no difference was noted between traditional LS, and the robotic device. However, by the final stages, RAS demonstrated less elbow extension and shoulder flexion. RULA demonstrated significantly less movement of the thumbs and fingers during RAS. |
| *Tokas et al.[42]* | Da Vinci Surgical System (Intuitive Systems, Sunnyvale, CA, USA) | Repeated-Measures | Questionnaire | Radical Prostatectomy (Training tasks) | Y | 7 | None of the participating surgeons experienced any ‘heavy’ (severe) discomfort during use of the Da Vinci system, compared to 80% in traditional LS, and between zero and 73% using ETHOS, across the three tasks. ETHOS demonstrated significant gains in ergonomy compared to traditional LS but still fell short of the Da Vinci system. |

**Supplementary Table 3.** Study characteristics of articles included in the review^[[1]](#footnote-1)^.

| Reference | Bias due to confounding | Bias in selection of participants into the study | Bias in classification of intervention | Bias due to deviation from intended intervention | Bias due to missing data | Bias in measurement of outcomes | Bias in selection of the reported result | Overall bias |
| --- | --- | --- | --- | --- | --- | --- | --- | --- |
| *Bagrodia et al.* | Moderate | Low | Low | Low | Low | Moderate | Low | Moderate |
| *Baumgarten et al.* | Low | Serious | Low | Low | Low | Low | Low | Moderate |
| *Bigham et al.* | Low | Low | Low | Low | Low | Moderate | Low | Low |
| Elhage *et al.* | Low | Moderate | Low | Low | Low | Moderate | Low | Moderate |
| Fan *et al.* | Low | Serious | Low | Low | Low | Moderate | Low | Moderate |
| Fiori *et al*. | Low | Moderate | Low | Low | Low | Moderate | Low | Moderate |
| Gaboardi *et al.* | No information | Moderate | Low | Low | Moderate | Moderate | Low | Moderate |
| Giberti *et al.* | No information | Low | Low | Low | Low | Moderate | Low | Moderate |
| Gofrit *et al.* | Low | Low | Low | Low | Low | Low | Low | Low |
| Hayashi *et al.* | Low | Low | Low | Low | Low | Low | Low | Low |
| Hubert *et al.* | Low | Low | Low | Low | Low | Low | Low | Low |
| Lee *et al.* | Low | Low | Low | Low | Low | Moderate | Low | Low |
| Marçon *et al.* | Moderate | Low | Low | Low | Moderate | Moderate | Low | Moderate |
| Norasi *et al.* | Moderate | Moderate | Low | Low | Low | Moderate | Low | Moderate |
| Park *et al.* | Low | Low | Low | Low | Low | Moderate | Low | Low |
| Pérez-salazar *et al.* | Low | No information | Low | Low | Low | Low | Low | Low |
| Rassweiler *et al.* | Low | Serious | Low | Low | Low | Low | Low | Moderate |
| Saglam *et al.* | Moderate | Low | Low | Low | Low | Moderate | Low | Moderate |
| Saikali *et al.* | Low | Moderate | Low | Low | Low | Moderate | Low | Moderate |
| Sánchez-Margallo *et al*. | Moderate | Low | Low | Low | Low | Low | Low | Low |
| Tokas *et al.* | Moderate | Low | Low | Low | Low | Moderate | Low | Moderate |

**Supplementary Table 4.** Risk of Bias in Non-Randomised Studies of Intervention (ROBINS-I) assessment of twenty-one included studies.

**
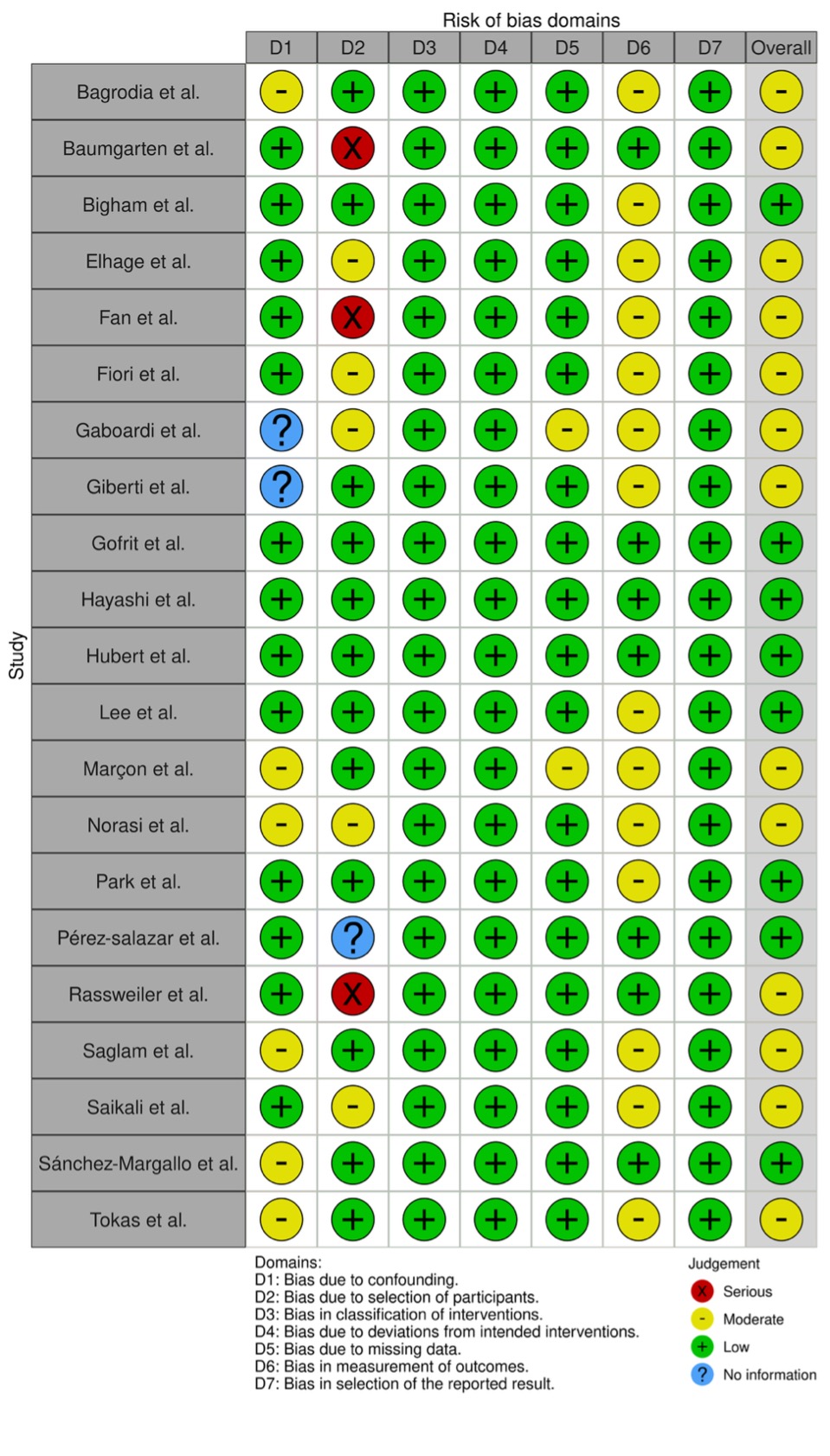
**

**
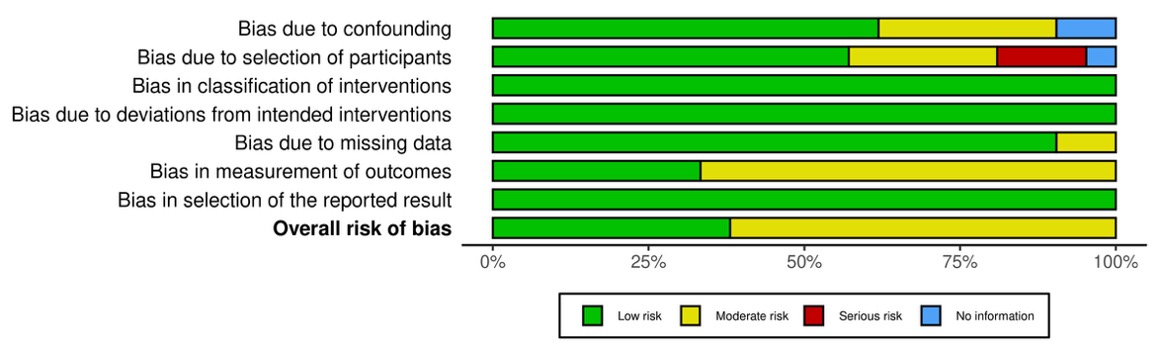
**

**Supplementary Figure 1.** ROBVIS risk of bias tool

1. **Abbreviations:** Robot-assisted surgery (RAS); Laparoscopic surgery (LS); Open Surgery (OS); Hand-assisted laparoscopic surgery (HALS); Electromyography (EMG); Subjective mental workload (SMW); Flexor carpi ulnaris (FCU); Biceps brachii (BB); Robotic laparoendoscopic single-site radical prostatectomy (R-LESS-RP); Robot-assisted radical prostatectomy (RARP); Rapid entire body assessment (REBA); Rapid upper limb assessment (RULA); NASA task load index (NASA-TLx); Surgeon’s task load index (SURG-TLx); Maximal voluntary contraction (MVC).

   **N(*):** Number of participating surgeons (number of urologists if multiple specialties investigated). [↑](#footnote-ref-1)
